# Supplementary material for: The Spatial Atlas of Human Anatomy (SAHA): A Multimodal Subcellular-Resolution Reference Across Human Organs
Source: bioRxiv. 2025 Jun 19:2025.06.16.658716. Preprint. [Version 2] doi: 10.1101/2025.06.16.658716 (PMC12224548; doi:10.1101/2025.06.16.658716)
Supplement: Supplement 1 [file NIHPP2025.06.16.658716v2-supplement-1.pdf]

bioRxiv preprint doi: https://doi.org/10.1101/2025.06.16.658716; this version posted June 19, 2025. The copyright holder for this preprint (which was not certified by peer review) is the author/funder, who has granted bioRxiv a license to display the preprint in perpetuity. It is made available under aCC-BY 4.0 International license.

SUPPLEMENTARY TABLES

Supplementary Table 1. SAHA Cohort Demographics and Clinical Metadata.
Summary of demographic and clinical characteristics of the SAHA cohort, including patient age, sex, tissue of origin, comorbidity (if applicable), and sample designation.

Supplementary Table 2. SAHA Experimental Run Metadata.
Detailed metadata for all SAHA spatial transcriptomics runs, including informations such as slide/sample identifiers, tissue types, assay panels, staining protocols, quality metrics/statistics, platform used (e.g., CosMx, Xenium), and batch/run identifiers.

Supplementary Table 3. Additional Information related to CRC Analysis.
Additional files regarding the CRC and COL comparative analysis including list of probe substitutes, differential gene expression lists, cell marker lists, full result list of Moran's I results.
